# Feasibility of paying people who use drugs cash to distribute naloxone within their networks

**DOI:** 10.1186/s12954-024-00947-6

**Published:** 2024-02-16

**Authors:** Nikki M. Lewis, Rebecca P. Smeltzer, Trevor J. Baker, Andrea C. Sahovey, Justine Baez, Erika Hensel, Brandon Poole, Cecelia Stewart, Allyson G. Cogan, Mackenzie Bullard, Jessica L. Taylor

**Affiliations:** 1 Berkshire Regional Planning Commission, Pittsfield, MA USA; 2 Department of Population and Public Health Sciences, Keck School of Medicine, University of Southern California, Los Angeles, CA USA; 3 Clinical Addiction Research and Education Unit, Section of General Internal Medicine, Department of Medicine, Boston Medical Center, Boston, MA USA; 4 ONESTOP Harm Reduction Center, Gloucester, MA USA; 5 Tapestry Health, Holyoke, MA USA; 6 Market Decisions Research, Portland, ME USA; 7 Grayken Center for Addiction, Boston Medical Center, Boston, MA USA; 8 Boston University, Chobanian and Avedisian School of Medicine, Boston, MA USA

**Keywords:** People who use drugs, Harm reduction, Naloxone

## Abstract

**Introduction:**

Immediate access to naloxone is needed to prevent fatal opioid-related overdoses in the presence of fentanyl analogs saturating the opioid supply. Peer models engage impacted populations who are not accessing naloxone through standard venues, yet compensating peers who utilize syringe service programs with cash stipends to distribute naloxone within networks of people who use drugs is not well described.

**Methods:**

As part of the HEALing Communities Study, syringe service program-based interventions were developed in Holyoke and Gloucester, MA, which paid people who use drugs (“peers”) cash to distribute naloxone. Early program outcomes were evaluated for the time each program was funded within the HCS study period.

**Results:**

During 22 study-months of observation, peers in two communities distributed 1104 naloxone kits. The total cost of peer compensation for program delivery was $10,510. The rate of peer-distributed naloxone per 100 K population reached 109 kits/mo and 222 kits/mo in the two communities. Participating peers addressed gaps in harm reduction outreach and distributed naloxone and other harm reduction equipment to individuals who were not syringe service program participants, expanding organizational reach. Being compensated with unrestricted cash stipends supported dignity and acknowledged peers’ work in overdose prevention.

**Conclusion:**

The underutilization of compensated peer models is often attributed to funding and organizational barriers. These programs demonstrate that providing cash stipends to peers is feasible and expanded naloxone distribution at two existing syringe service programs. Providing cash stipends for peers who engage in secondary naloxone distribution offers promise in delivering naloxone to people not accessing syringe services.

## Introduction

Fatal opioid-related overdoses are a public health emergency in the United States (US), resulting in more than 80,000 deaths in 2022, the majority due to synthetic opioids, excluding methadone, such as illicitly manufactured fentanyl [1, 2]. Fentanyl analogs, which saturate the opioid supply nationally, drive overdose deaths due to high, unpredictable potency, rapid onset, and overdose events that can progress over seconds to minutes. Naloxone is an opioid receptor antagonist that can reverse opioid-related overdose. Studies of Overdose Education and Naloxone Distribution programs demonstrate that the annual distribution of 100–250 naloxone kits per 100,000 population to people who use drugs, and potential overdose bystanders is associated with a 46% reduction in community opioid overdose mortality [3].

While naloxone remains effective in the setting of fentanyl-involved overdose and substantial progress has been made in making naloxone available in commercial pharmacy, clinical, and harm reduction settings in the US and internationally, the distribution of naloxone kits is most effective when distributed directly to people who use drugs at high risk of opioid-related overdose, through programs such as syringe service programs, emergency department distribution, and street outreach [4, 5]. Novel strategies for naloxone distribution are urgently needed to ensure that naloxone is immediately available, at the point of substance use, to people who use drugs who do not access naloxone through standard venues due to barriers such as stigma, transportation, criminalization, poverty and mistrust [6–8].

Peer models are important tools that may increase the influence and autonomy of systematically marginalized groups [6, 9–12]. In this context, the term “peers” is used to describe people who currently use substances, which uniquely informs their work in overdose prevention [6]. Peer overdose prevention models are underutilized; when implemented, “token” or inadequate compensation for time and expertise has been described as undermining effectiveness [6, 9]. Insufficient training, supervision, and mentorship for peers have also been barriers in prior models [6, 9].

The goal of this paper is to describe the feasibility of using existing syringe service program infrastructure to pay people who use drugs cash, as opposed to more restrictive compensation methods such as gift cards, to distribute naloxone to people at high risk of overdose within their social networks. We evaluate early naloxone distribution and other program outcomes.

## Methods

The HEALing Communities Study (HCS) is a NIH-funded, multisite, randomized controlled trial aimed at reducing opioid overdose deaths through the Communities that HEAL (CTH) intervention [13]. To increase naloxone distribution, HCS coalitions in Holyoke and Gloucester, Massachusetts supported local syringe service programs to compensate peers with cash stipends to distribute naloxone kits directly to other people who use drugs. Each naloxone kit distributed contained two doses of naloxone 4 mg/0.1 mL nasal spray. Within this model, peers receive payments to obtain naloxone kits and, in some cases, other harm reduction supplies from a syringe service program and distribute them among their social networks, engaging people who experience barriers accessing brick-and-mortar or mobile syringe service program sites [14].

Both communities identified that in order for these programs to be successful, formal contracting and Criminal Offender Record Information would not be required, and unrestricted cash stipends would be utilized as payment to advance equity and rightfully compensate peers for their overdose prevention work. In Holyoke, the syringe service program had a longstanding “cash on hand” fund maintained for small expenses. They were able to pay peers through this fund without significant operational changes. Conversely, the Gloucester syringe service program did not have an existing pathway to pay peers in cash. Developing a new protocol for cash payments involved advocacy with organizational leadership regarding the benefits of cash payments, consultation with the organization’s finance team, and development of appropriate accounting practices including dual signoff on all cash payments by both a manager and the peer distributor.

Program impact was evaluated using descriptive statistics and narrative information from monthly data reporting, along with HCS coalition meeting minutes, to identify key program themes.

The Holyoke and Gloucester HCS coalitions approved the peer naloxone distribution interventions in December 2020 and October 2021, respectively. Syringe service program staff recruited peers from their programs, focusing on participants who frequented the brick-and-mortar locations and demonstrated a commitment to overdose prevention within their communities. Interventions launched in March 2021 in Holyoke and January 2022 in Gloucester. Table 1 details the program similarities and differences.

**Table 1:** Description of peer naloxone distribution programs in Holyoke and Gloucester, Massachusetts, 2021–2022

Program Components	Holyoke	Gloucester
Implementing syringe service program	Tapestry Health	ONESTOP HRC
Duration of HCS funding	March 2021–June 2022 (16 months)	January 2022–June 2022 (6 months)
Program manager	Harm reduction specialist	Harm reduction specialist
Identification of peers	Pre-approved list based on peer interest	Direct outreach to engage peers
Duration of peer participation	Four consecutive weeks, with option to reenroll	No time limit
Weekly compensation per peer	Up to $25 ($5 per naloxone kit), cash	$125, cash
Supplies distributed	Naloxone kits	Naloxone kits and other harm reduction supplies*
Average program cost per month	$219	$1167
Total program cost	$3510	$7000

*Other harm reduction supplies included fentanyl test strips, safer injection equipment, safer smoking and snorting kits, sharps disposal containers, condoms, and syringes which were both distributed and collected for disposal and supported by funding outside of the HCS

Peers documented the number of naloxone kits distributed, the distribution location (general), and, in the case of the Gloucester program, additional harm reduction supplies exchanged. Peers and syringe service program staff regularly discussed program challenges, successes, and peer suggestions to improve outcomes.

## Results

Peers in Holyoke and Gloucester distributed 1104 naloxone kits in total, averaging 56 kits per month (Table 2). In Holyoke, most peers identified as people experiencing homelessness, spoke a non-English primary language, and/or engaged in sex work, which provided them with connections to these communities for harm reduction outreach and service delivery outside of traditional brick-and-mortar syringe service program locations. The rotating schedule built into Holyoke’s peer naloxone distribution program was also intentionally designed to encourage the participation of many different people who use drugs, which expanded the reach of this strategy based on each peer distributor’s unique personal and professional social networks. From March 2021 through June 2022, peers accounted for 16% of the Holyoke syringe service program’s naloxone distribution, averaging 44 kits per month. The highest number of naloxone kits distributed in a month by Holyoke peers was 85 kits.

**Table 2:** Peer naloxone kit distribution in Holyoke and Gloucester, Massachusetts, 2021–2022

Program Outcomes	Holyoke (Tapestry) Mar 2021–Jun 2022	Gloucester (ONESTOP HRC) Jan 2022–Jun 2022
Average number of peer distributors, monthly	5	2
Naloxone kits distributed by syringe service programs excluding peer distribution	3785	1157
Naloxone kits distributed by peers	702	402
Highest monthly number of naloxone kits distributed	85	148
Lowest monthly number of naloxone kits distributed	10	10
Rate of naloxone distribution by peers	109 naloxone kits/100,000 residents	222 naloxone kits/100,000 residents

In Gloucester, an average of two peers per month distributed naloxone and harm reduction supplies. Peers had differing community connections, one experiencing homelessness and one with ties to the commercial fishing community. The commercial fishing community experiences high rates of overdose, specific pressures to conceal substance use, and barriers to treatment [15]. From January through June 2022, peers accounted for 26% of the Gloucester syringe service program’s total naloxone distribution, averaging 67 kits per month. Through funding outside of the HCS, peers distributed 138 fentanyl test strips, 40 sharps containers, 18 safer smoking kits, 16 safer snorting kits, 101 condoms, and 3568 syringes, and they collected 2902 syringes for safe disposal.

The narrative data provided by syringe service program staff and peers described ways in which peer naloxone distributors addressed gaps in harm reduction outreach in both communities. For example, during months in which Holyoke staff reported COVID-19-related staff shortages the peer stipend program was able to continue, and the syringe service program’s overall naloxone distribution remained stable or increased. In Gloucester, staff reported in April 2022 that as weather began to warm, populations began moving around the area, which created challenges in making connections through traditional outreach methods. However, in the same month, peers distributed more naloxone than they did in any other month.

## Discussion

Amidst the highest rates of opioid overdose deaths in US history, peers are uniquely positioned to expand access to naloxone to those at highest risk. The results demonstrate that syringe service program-based peer naloxone distribution models that utilize cash compensation are feasible and associated with an increase in naloxone distribution. Anecdotally, peers’ unique community connections extended the syringe service program’s reach to community members who may not access harm reduction services through traditional stationary or mobile venues. Peers also reported that engagement in the program was a source of community, especially when facing the isolation that may accompany responding to overdoses. One peer utilized program connections to secure long-term employment and assisted other peers in obtaining per diem roles.

Prior work in Massachusetts demonstrated that annual coverage of 100–250 naloxone kits per 100,000 residents was associated with a 46% reduction in opioid overdose death rate [3]. Although our study was not designed to evaluate impacts on mortality, the rate of naloxone distribution achieved by the peer models is high enough to yield potentially meaningful reductions in opioid overdose fatalities. Furthermore, the benefits of a peer-led model targeting people who use drugs who are unwilling or unable to access syringe service program services may exceed the benefits observed in prior studies due to the intentional delivery to people who use drugs at very high risk. Future work should evaluate the impact of peer naloxone distribution on downstream outcomes, including opioid overdose fatality.

The peer naloxone distribution programs were low-cost, amounting to $10,510 in total for both programs and averaging $11 per naloxone kit distributed. These funds covered the cost of compensation for the peer distributors who received $5 per naloxone kit distributed, up to $25 per week, in Holyoke and $125 per week in Gloucester. The naloxone itself was provided at no cost to these programs through the Massachusetts Department of Public Health. Program delivery through the existing syringe service program infrastructure supported cost-effectiveness and feasibility. Both programs filled positions easily and had low turnover indicating high interest in this role. However, despite feasibility, securing long-term funding for non-billable public health interventions remains a challenge. Holyoke secured funding through 2026 via federal COVID-19 relief funds, while Gloucester continues to seek long-term funding. Other organizations may encounter additional barriers to providing cash compensation based on their organization and region-specific policies and procedures. Additional flexibility of existing public health funding and novel funding sources are necessary to scale cash payments for peer naloxone distribution.

## Limitations

This study has several limitations. First, its location in Massachusetts, a state with significant public infrastructure for harm reduction, and at two well-established syringe service programs with robust community relationships and trust among people who use drugs may limit generalizability to settings with less developed harm reduction infrastructure or more restrictive syringe service regulations. Both implementing organizations are state-funded Overdose Education and Naloxone Distribution programs that receive funding through the Massachusetts Department of Public Health and are therefore able to receive naloxone from the state and provide it to the community at no cost, which may be unavailable in other states. These interventions were delivered as part of a large community-engaged overdose prevention study with significant implementation support and thus may be harder to launch in syringe service programs with fewer resources. Furthermore, the study did not collect demographic information from individuals who received naloxone from peer distributors. Future interventions can consider the benefits of additional data collection, including in evaluating equity in intervention delivery and concurrent engagement in traditional syringe service programs, balancing privacy considerations and the barriers posed by additional administrative requirements.

Future evaluations of peer naloxone distribution models should incorporate qualitative interviews with peers and individuals who receive naloxone from peers to better understand barriers and facilitators of effective naloxone distribution in this model and potential non-monetary benefits of peer participation, including developing a record of employment and professional references.

## Conclusion

Providing cash stipends to people who use drugs to distribute naloxone is a feasible method to extend the reach of syringe service programs and reach those at high risk of overdose who are not already engaged in harm reduction services. People who use drugs have a long history of protecting one another, including in preventing opioid overdose. Investing in our communities to increase the capacity for naloxone distribution within networks of people who use drugs via appropriate cash compensation for their time and expertise offers promise in addressing our current crisis.

## Data Availability

The datasets generated and/or analyzed during the current study are not publicly available due the small sample size and privacy of participating individuals but are available from the corresponding author on reasonable request.
